# Dynamics of Transposable Element Invasions with piRNA Clusters

**DOI:** 10.1093/molbev/msz079

**Published:** 2019-04-09

**Authors:** Robert Kofler

**Affiliations:** Institut für Populationsgenetik, Vetmeduni Vienna, Wien, Austria

**Keywords:** transposable elements, piRNA clusters, evolutionary dynamics, population genetics, transposon invasions, forward simulations

## Abstract

In mammals and invertebrates, the proliferation of an invading transposable element (TE) is thought to be stopped by an insertion into a piRNA cluster. Here, we explore the dynamics of TE invasions under this trap model using computer simulations. We found that piRNA clusters confer a substantial benefit, effectively preventing extinction of host populations from a proliferation of deleterious TEs. TE invasions consist of three distinct phases: first, the TE amplifies within the population, next TE proliferation is stopped by segregating cluster insertions, and finally the TE is inactivated by fixation of a cluster insertion. Suppression by segregating cluster insertions is unstable and bursts of TE activity may yet occur. The transposition rate and the population size mostly influence the length of the phases but not the amount of TEs accumulating during an invasion. Solely, the size of piRNA clusters was identified as a major factor influencing TE abundance. We found that a single nonrecombining cluster is more efficient in stopping invasions than clusters distributed over several chromosomes. Recombination among cluster sites makes it necessary that each diploid carries, on the average, four cluster insertions to stop an invasion. Surprisingly, negative selection in a model with piRNA clusters can lead to a novel equilibrium state, where TE copy numbers remain stable despite only some individuals in a population carrying a cluster insertion. In *Drosophila melanogaster*, the trap model accounts for the abundance of TEs produced in the germline but fails to predict the abundance of TEs produced in the soma.

## Introduction

Transposable elements (TEs) are short stretches of DNA that selfishly multiply within genomes, even when this activity has deleterious effects to the host (Doolittle and Sapienza 1980; Orgel and Crick 1980). Deleterious effects may arise by three distinct mechanisms: 1) TE insertions could directly disrupt genes or promoter regions, 2) ectopic recombination between insertions at different sites could lead to deleterious genomic rearrangements, and 3) the products of TEs such as the *Transposase* could be deleterious (e.g., by generating DNA damage as found during hybrid dysgenesis) (Nuzhdin 1999; Moon et al. 2018). However, also several beneficial TE insertions, for example, conferring resistance to insecticides, have been identified (Aminetzach et al. 2005; Casacuberta and González 2013). Overall, the fitness cost of TEs remains controversial. A recent review therefore argued that the null hypothesis for the fitness consequences of TE insertions should be the neutral model (i.e., a TE insertions have no or little effect on host fitness) (Arkhipova 2018).

Due to the ability to proliferate within genomes, TEs frequently invade novel populations and species (Kidwell 1983; Kofler, Hill, et al. 2015; Peccoud et al. 2017). There is ample evidence that an invasion of a TE may be triggered by horizontal transfer from a distant species (Kidwell 1983; Montchamp-Moreau 1990; Rozhkov et al. 2013; Kofler, Hill, et al. 2015). It is likely that an invasion may also be triggered by processes that reactivate dormant TEs, such as environmental and genomic stresses, and by mutations within genes suppressing TE activity (McClintock 1984; Prud’homme et al. 1995; Capy and Gibert 2004; Sarot et al. 2004; Kalmykova et al. 2005; Beauregard et al. 2008; Wylie et al. 2016). Irrespective of what triggered an invasion, an unchecked proliferation of TEs may drive host populations extinct (Brookfield and Badge 1997), it is thus essential for the organism to control the spread of TEs. It was long thought that the proliferation of TEs is counteracted at the population level by natural selection acting against deleterious TE insertions (Charlesworth and Charlesworth 1983; Charlesworth and Langley 1989; Barrón et al. 2014). According to this “transposition-selection balance model,” TE copy numbers within a population are at an equilibrium between transposition events generating new insertions and negative selection removing insertions (Charlesworth and Charlesworth 1983; Charlesworth and Langley 1989; Barrón et al. 2014).

However, the discovery of the small RNA–based defense system profoundly changed our view on TE dynamics. It showed that the spread of TEs is not solely counteracted at the population level but actively combated by the host (Lee and Langley 2010; Blumenstiel 2011). The host defense system relies on the so called piRNAs, small RNAs ranging in size from 23 to 29 nt (Brennecke et al. 2007; Gunawardane et al. 2007). piRNAs bind to PIWI-clade proteins and mediate the suppression of TEs at the transcriptional and at the posttranscriptional level (Brennecke et al. 2007; Gunawardane et al. 2007; Sienski et al. 2012; Le Thomas et al. 2013). piRNAs are largely derived from discrete genomic loci that have been termed piRNA clusters (Brennecke et al. 2007; Malone et al. 2009). These piRNA clusters are frequently found in the heterochromatin, close to the euchromatin boundary, and may make up a substantial fraction of genomes (Brennecke et al. 2007). For example, in *Drosophila melanogaster*, piRNA clusters constitute about 3.5% of the genome (Brennecke et al. 2007). Several studies found that a single TE insertion in a piRNA cluster may be sufficient for repressing the activity of a TE (Ronsseray et al. 1991; Josse et al. 2007; Zanni et al. 2013). Such observations gave rise to the “trap model,” which holds that an invading TE proliferates within a host until at least one copy jumps into a piRNA cluster (the trap), which triggers production of piRNAs that silence the invading TE (Bergman et al. 2006; Malone and Hannon 2009; Zanni et al. 2013; Goriaux et al. 2014; Yamanaka et al. 2014; Ozata et al. 2019).

Interestingly, TEs may employ different strategies to increase in copy numbers (Blumenstiel 2011). They may either be active directly in the germline or in the somatic tissue surrounding the germline (henceforth “germline TEs” and “somatic TEs,” respectively). Somatic TEs usually require virus like particles to infect the germline (Song et al. 1997). Notably, these two different groups of TEs may be controlled by two different specialized piRNA pathways that rely on distinct sets of piRNA clusters (Li et al. 2009; Malone et al. 2009). These two sets of piRNA clusters may further have distinct architectures (Li et al. 2009; Malone et al. 2009). In *D. melanogaster*, somatic TEs are controlled by a single piRNA cluster, *flamenco*, which is located in heterochromatic regions of the X-chromosome, whereas germline TEs are controlled by several piRNA clusters distributed over multiple chromosomes (Brennecke et al. 2007; Malone et al. 2009). Additionally, TE insertions in *flamenco* are overwhelmingly in an antisense orientation, whereas no such bias was found for insertions in germline clusters (Malone et al. 2009).

piRNAs and piRNA clusters have been found in many different species such as flies, worms, mouse, and humans (Aravin et al. 2007; Yamanaka et al. 2014; Czech and Hannon 2016; Lewis et al. 2018). It is therefore likely that the trap model holds for most invertebrates and mammals. Despite the wide applicability, few theoretical studies explored the dynamics of TE invasions under the trap model. Kelleher et al. (2018) found that TE invasions are initially stopped by segregating cluster insertions and that the size of piRNA cluster influences the amount of TEs accumulating during an invasion. Lu and Clark (2010) found that piRNA clusters lower the fitness cost of TE insertions. Both studies found that TE insertions in piRNA clusters may be positively selected (Lu and Clark 2010; Kelleher et al. 2018). Other theoretical works investigated the equilibrium distribution of TEs in populations with (Charlesworth and Charlesworth 1983; Kaplan and Brookfield 1983; Langley et al. 1983) and without recombination (Sawyer and Hartl 1986; Moody 1988; Basten and Moody 1991; Morel et al. 1993), the dynamics of families that regulate their own activity (autoregulation) (Charlesworth and Langley 1986; Townsend and Hartl 2000; Bouuaert et al. 2013), the frequency distribution of nonautonomous TEs (Kimmel and Mathaes 2010), the influence of the breeding system on TE dynamics (Wright and Schoen 1999), the fate of TEs during early stages of an invasion (Le Rouzic and Capy 2005; Marshall 2008a, 2009), the spread of internally deleted TEs during invasions (Marshall 2008b) and the long-term coevolution between TEs and their hosts (Le Rouzic et al. 2007).

To gain more insights into TE invasions with piRNA clusters, we performed large-scale simulations of TE invasions under the trap model using our novel simulator Invade (https://sourceforge.net/projects/invade/; last accessed April 18, 2019). We show that piRNA clusters are highly beneficial to host populations as they prevent extinction from an uncontrollable proliferation of deleterious TEs. We furthermore show that TE invasions have three distinct phases. We found that the size of piRNA clusters is the most important factor governing the amount of TEs accumulating during an invasion and that the somatic architecture is more efficient in stopping invasions than the germline architecture. Finally, using publicly available data from *D. melanogaster*, we found that the trap model reasonably well accounts for the abundance of germline TEs but fails to explain the abundance of somatic TEs.

## Results

**Fig. 1.**
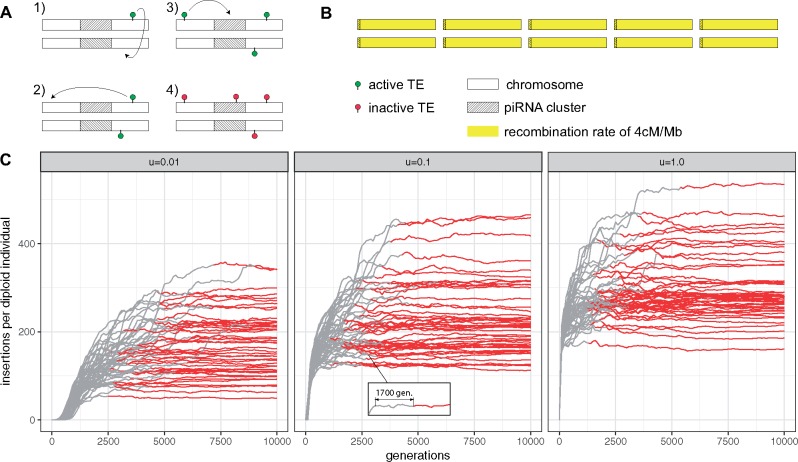
piRNA cluster may stop TE invasions. (*A*) Under the trap model, an active TE (green) multiplies within the genome (rectangles indicate chromosomes of a diploid organism) until one copy jumps into a piRNA cluster (i.e., the trap, hatched area) whereupon all TEs, including those on homologous chromosomes, get inactivated in trans (red). A heterozygous insertion is sufficient to suppress all TEs (dominant effect). (*B*) We simulated five chromosomes of size 10 Mb for a diploid organism. Each chromosome carried a piRNA cluster of size 300 kb. A constant recombination rate of 4 cM/Mb (yellow) was used. (*C*) Abundance of TEs during an invasion. Populations of size *N *=* *1,000 and neutral TE insertions were simulated. We show 50 replicates for three different transposition rates (*u*: top panel). All populations eventually acquired a fixed cluster insertion (red line), which permanently inactivates the TE. Negative selection against TEs is thus not necessary to stop TE invasions under the trap model.

The trap model holds that proliferation of an invading TE is stopped by a random insertion into a piRNA cluster (fig. 1*A*). In this work, we performed large-scale simulations to gain a deeper understanding of the population dynamics of TE invasion under the trap model. We simulated five chromosomes with a size of 10-Mb, a recombination rate of 4 cM/Mb, and a piRNA cluster of size 300 kb at the beginning of each chromosome (fig. 1*B*). Thus, similarly as in *Drosophila*, the total size of the piRNA cluster accounts for 3% of the genome (Brennecke et al. 2007). Per default, we used a population size of *N *=* *1,000. We launched TE invasions by randomly distributing ten insertions in individuals of the starting population. Since all TEs in the starting population segregate at low frequency (1/2N), it is feasible that TE may be lost in a population due to genetic drift (Le Rouzic and Capy 2005). For small transposition rates (*u*), the probability of losing a TE insertion with frequency 1/2N is approximately $p_{l}=1-2u$ (where $p_{l}\geq 0$) (Le Rouzic and Capy 2005). Hence, the probability of successfully establishing a TE invasion is $p_{e}=1-{\left(p_{l}\right)}^{n}$ with *n* being the number of insertions in the starting population. Using, for example, the different transposition rates *u *=* *0.01, *u *=* *0.1, and *u *=* *1.0, we obtain probabilities of establishments of $p_{e}=0.183,\;p_{e}=0.893$, and $p_{e}=1.0$, respectively (*n *=* *10). Our simulations agree with this expectation. Out of 1,000 simulations, the invasion got established in 168, 803 and 1,000 replicates which is close to theoretical expectations (183, 893 and 1,000, respectively; 500 generations were simulated). Since we are mainly interested in the dynamics of successful TE invasions, we henceforth ignore TE invasion that failed to establish (unless mentioned otherwise).

Classic population genetic models (transposition-selection balance), developed before the discovery of piRNAs, show that the proliferation of TEs can be contained by negative selection against TEs (Charlesworth and Charlesworth 1983; Charlesworth and Langley 1989). We first tested the hypothesis that piRNA clusters are capable of containing the spread of TEs in the absence of negative selection against TEs. We simulated 100 TE invasions for 20,000 generations using three different transposition rates (*u *=* *0.01, *u *=* *0.1, and *u *=* *1.0; fig. 1*C*). Initially, we simulated neutral TE insertions (i.e., TE insertions have no fitness costs to the host). Negative selection against TEs is treated later. Here, we define a TE invasion to be “stopped” once a cluster insertion gets fixed, that is, a cluster insertion at a particular genomic site reaches a frequency of 1.0. A fixed cluster insertion permanently inactivates the TE. By generation, 20,000 all replicates for each transposition rate acquired at least one fixed cluster insertion (trajectories for 50 replicates and 10,000 generations are shown in fig. 1*C*). In contrast to this finding, Kelleher et al. (2018) found that cluster insertions rarely get fixed. This discrepancy is likely due to the smaller number of generations used by Kelleher et al. (2018) (500 generations vs. 20,000 in this work). We conclude that the piRNA clusters are able to stop TE invasions, even when transposition rates are extremely high and TE insertions are neutral (fig. 1*C*).

**Fig. 2.**
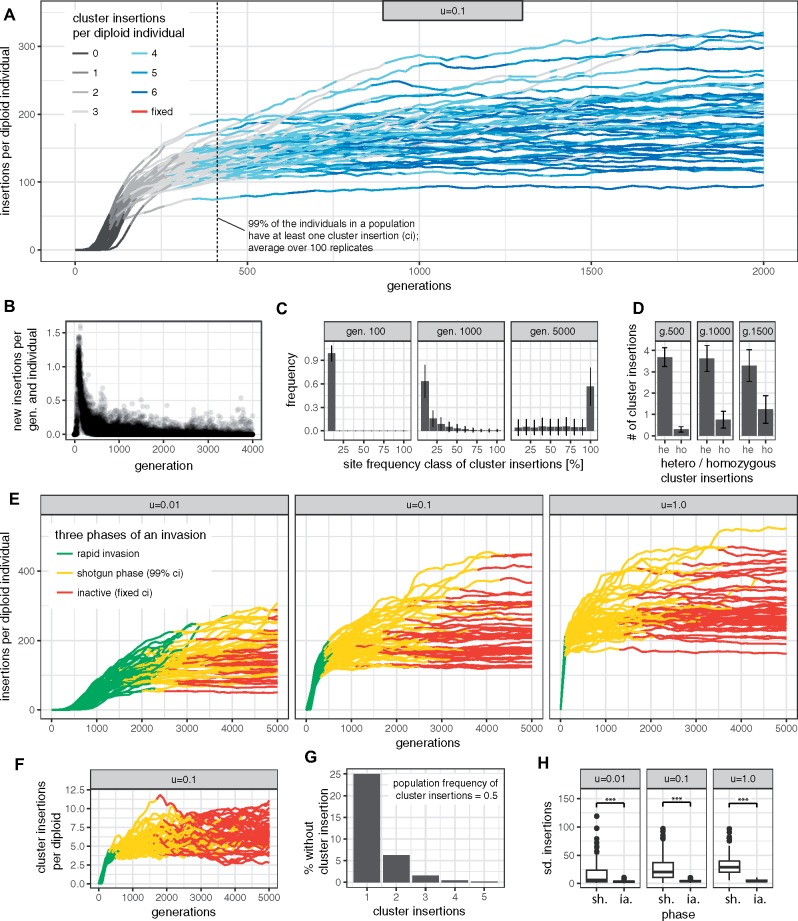
TE invasions consist of three distinct phases. (*A*) Under the trap model, TE invasions are initially stopped by multiple segregating cluster insertions. The invasions slow down as the number of cluster insertions per individual increases. Dashed line indicates the generation at which on the average >99% of the individuals within a population acquired at least one cluster insertion. No fixed cluster insertions were observed by generation 2,000. (*B*) Number of novel insertions per individual during TE invasions. (*C*) Site frequency spectrum of cluster insertions during TE invasions. At early stages of an invasion (e.g., generations ≤1,000), all cluster insertions segregate at low frequency. Error bars indicate standard deviation based on 100 replicates. (*D*) Fraction of homo- (ho) and heterozygous (he) cluster insertions at different generations (g). (*E*) The three phases of TE invasions for different transposition rates (*u*). Fifty replicates are shown. (*F*) Number of cluster insertions for the three phases of TE invasions. (*G*) Fraction of individuals without cluster insertions (i.e., with an active TE), dependent on the number of segregating cluster insertions. (*H*) Stability of phases measured in standard deviation (sd.) of TE copy numbers. The shotgun phase (sh.) is significantly less stable than the inactive phase (ia.; ***P <* *0.01).

We noticed that in some replicates TE copy numbers stabilized for many hundred generations despite no cluster insertion being fixed (fig. 1*C* inlay), which suggests that the TE invasion may be contained by segregating cluster insertions, as proposed previously (Kelleher et al. 2018; Kofler et al. 2018). We therefore investigated the early stages of TE invasions in more detail. Interestingly, we found that TE copy numbers plateaued in all replicates although no cluster insertion got fixed (fig. 2*A*;*u *=* *0.1). The average amount of novel TE insertions per generation and individual significantly decreased from 1.001 at generation 100 to 0.043 at generation 500 (Wilcoxon rank sum test; *W *=* *9,895, P<2.2e−16; fig 2*B*). This plateauing of the invasion was accompanied by an increase in the average amount of cluster insertions per individual, from 0.85 at generation 100 to 5.12 at generation 500 (Wilcoxon rank sum test; *W *=* *0, P<2.2e−16; fig 2*A*). At early stages of the invasions, all cluster insertions segregate at a low frequency, whereas high frequency insertions emerge at later stages (fig. 2*C*;supplementary fig. 3, Supplementary Material online). Accordingly, most cluster insertions were heterozygous at early stages of the invasions (fig. 2*D*). Our results thus support the view that TE invasions are initially stopped by segregating cluster insertions. However, at later stages, fixed cluster insertions emerge (fig. 2*C*; generation 5,000) which inactivate the invading TE (fig. 2*B*). Hence, our results suggest that TE invasions under the trap model consist of three distinct phases. First, TE copy numbers rapidly increase fairly unconstrained. We termed this stage as the “rapid invasion” phase (fig. 2*E*, green). Second, TE invasions are contained by segregating cluster insertions. Consistent with our previous work, we term this stage the “shotgun phase,” to signify that cluster insertions are widely distributed over many distinct genomic sites (fig. 2*E*, yellow [Kofler et al. 2018]). Delimiting the exact onset of the shotgun phase is however a bit arbitrary. In this work, we use the moment at which 99% of the individuals acquired at least one cluster insertion as the onset of the shotgun phase. Invasion considerably slowed down at this stage (fig. 2*A*; dashed line). Third, fixation of a cluster insertions leads to a complete inactivation of the TE. Hence, we termed this stage the “inactive” phase (fig. 2*E* red). We found that the number of insertion sites in the population increased sharply during the rapid invasion phase, decreased slowly during the shotgun phase and stabilized in the inactive phase (supplementary fig. 2, Supplementary Material online). The site frequency spectrum of cluster and noncluster insertions was identical during the invasion (supplementary fig. 3 and table 1, Supplementary Material online). Hence, cluster insertions are not positively selected when TE insertions have no impact on host fitness.

Interestingly, we observed that at the onset of the shotgun phase each individual had on the average acquired 3.8 cluster insertions (e.g., with *u *=* *0.1; fig. 2*F*), although a single insertion would have been sufficient to silence the TE. This result can be explained by the fact that cluster insertions are segregating. Assume a scenario where a single cluster insertion has a population frequency of 0.5. Due to Hardy–Weinberg equilibrium, 25% of the individuals will not have a cluster insertion. Extending this example to two cluster insertions at distinct genomic sites, then of the 25% of individuals without cluster insertion at the first locus, another 25% will not have an insertion at the second locus. The TE will thus be active in the 6.25% of individuals without cluster insertion (fig. 2*G*). The fraction of individuals with an active TE can thus be computed as $f_{a}=\prod_{i}{\left(1-p_{i}\right)}^{2}$ where *p_i_* is the population frequency of the *i*th cluster insertion. Our data suggest that on the average 3.8 cluster insertions per diploid are necessary to reduce the fraction of individuals with an active TE sufficiently such that TE copy numbers stagnate.

We noticed that in some replicates TE copy numbers increased abruptly during the shotgun phase (fig. 2*E*). To quantify the stability of the phases, we computed the standard deviation of the TE abundance (population mean) during each phase for every replicate separately (fig. 2*H*). We found that TE abundance during the shotgun phase is significantly less stable than during the inactive phase (Wilcoxon rank sum test for *u *=* *1, *u *=* *0.1, and *u *=* *0.01; each P<4.1e−08; fig. 2*H*). Our results thus suggest that silencing of TE invasion by segregating cluster insertions is unstable. Solely, fixation of a cluster insertions results in permanent inactivation of the TE and thus in stable TE copy numbers.

**Fig. 3.**
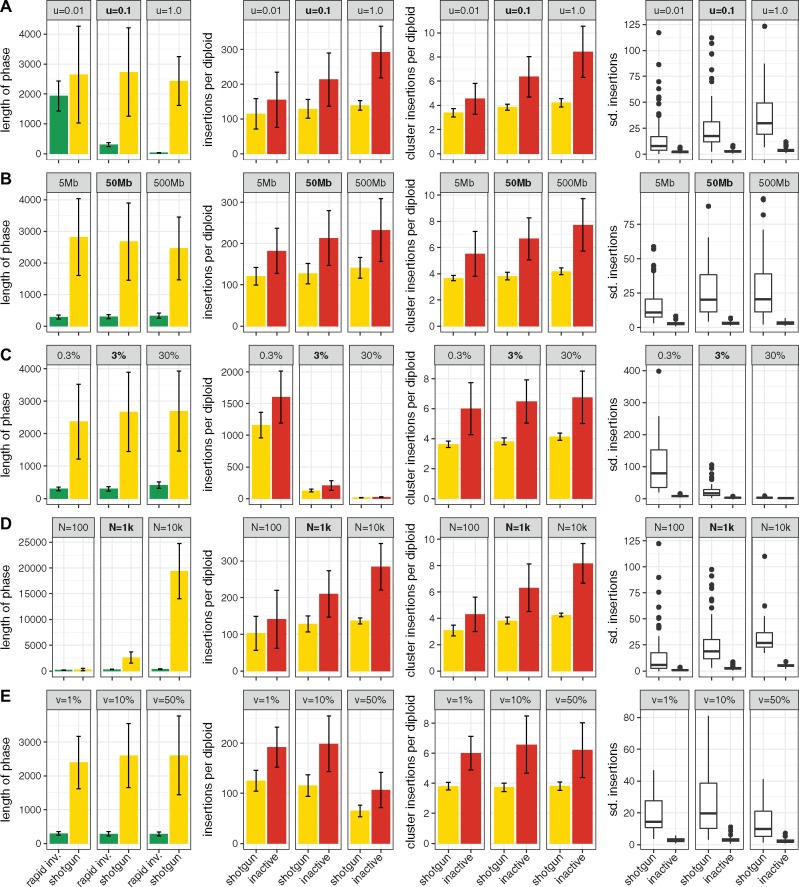
Influence of different factors on TE invasions. We studied the influence of the transposition rate (*A*), the genome size (*B*), the size of piRNA clusters, in percent of the genome (*C*), the population size (*D*), and the excision rate (*E*). We used default parameters (bold) for the simulations and varied solely the factor of interest (for excisions the default is v=0.0%). We show the impact of the different factors on the length of the phase (in generations), the TE abundance per diploid individual at the start of the phase, the number of cluster insertions per diploid individual at the start of the phase and the stability of phase measured in standard deviation of the TE abundance (sd. insertions).

Next, we asked which factors influence the dynamics of TE invasion under the trap model. We evaluated the impact of the transposition rate (*u*), the genome size, the size of the piRNA clusters (in percent of the genome size), the population size (*N*), and the excision rate (*v*). To minimize the parameter space for the simulations, we used default conditions (*u *=* *0.1, genome size = 50 Mb, cluster size = 3%, *N *=* *1,000, and v=0%) and varied only the parameter of interest within these defaults (fig. 3; defaults are shown bold). We assessed the impact of these factors on the following key properties of invasions: the length of the phase, the TE abundance at the beginning of the phase, the abundance of cluster insertions at the beginning of the phase, and the stability of the phase (quantified as standard deviation of the TE abundance per phase and replicate). We omitted meaningless or irrelevant data such as the length of the inactive phase (infinite) or the TE abundance at the beginning of the rapid invasion phase (10/(2*N)) (fig. 3). We found that the transposition rate had a strong influence on the length of the rapid invasion phase but little influence on other properties, including the abundance of TE insertions (fig. 3*A*;supplementary table 2, Supplementary Material online). This result is notable as the transposition rate is a major factor governing TE abundance under the transposition-selection balance model (Charlesworth and Charlesworth 1983; Kofler, Nolte, et al. 2015). As expected, the genome size had very little influence on the invasion dynamics (fig. 3*B*;supplementary table 2, Supplementary Material online). The reason why it had any influence at all may be that we ignored insertions into already occupied sites. Such double insertions are more likely to occur in smaller genomes where fewer TEs will accumulate as a consequence. The size of the piRNA clusters had an enormous influence on the number of TEs accumulating during an invasion, where most TEs were found for small clusters (fig. 3*C*;supplementary table 2, Supplementary Material online). For small clusters, many more insertions will be necessary until one copy randomly jumps into a piRNA cluster. This finding is in agreement with Kelleher et al. (2018) who also found an influence of the cluster size on TE abundance. Interestingly, the population size influenced the length of the shotgun phase, where larger populations have longer shotgun phases (fig. 3*D*;supplementary table 2, Supplementary Material online). Genetic drift is weak in large populations. Hence, fixation of cluster insertions, which marks the end of the shotgun phase, will require more time. Due to this longer duration of the unstable shotgun phase, more TEs will accumulate in large populations (fig. 3*D*). Note that this result is in stark contrast to the classic transposition-selection balance model, where fewer TEs are expected to accumulate in large populations as the efficacy of negative selection against TEs is higher in large populations (Charlesworth and Charlesworth 1983; Kofler, Nolte, et al. 2015). The excision rate only had a small influence on invasion dynamics (fig. 3*E*;supplementary table 2, Supplementary Material online). With our model, excisions from piRNA clusters are not feasible as we assume that TEs are inactive (transpositions as well as excisions) in individuals with a cluster insertion. Also, the recombination rate only had a weak influence on invasion dynamics (supplementary fig. 4 and table 2, Supplementary Material online). Surprisingly, we found that irrespective of the simulated scenario always about four to six cluster insertions per diploid where necessary to stop the invasions (fig. 3). piRNA clusters should thus contain multiple insertions from silenced families.

**Fig. 4.**
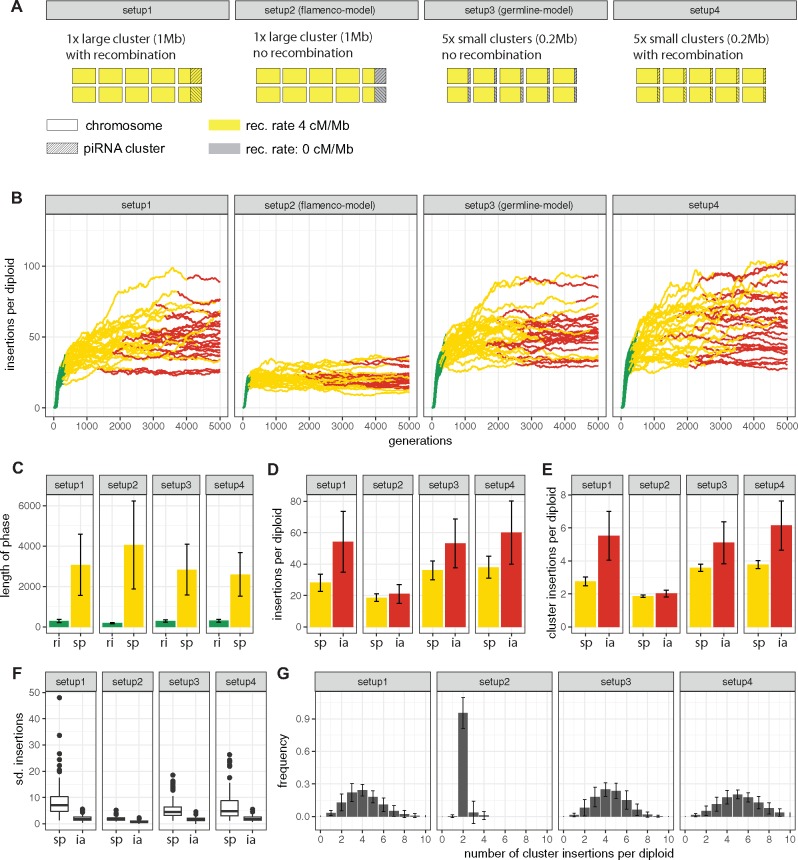
Influence of piRNA cluster architecture on TE invasions. (*A*) Overview of the simulated architectures. Using *Drosophila* as example, the single nonrecombining cluster resembles the architecture of the *flamenco* locus (setup 2), whereas multiple nonrecombining clusters resemble the germline piRNA clusters (setup 3). (*B*) TE abundance during invasions for the different architectures. Fifty replicates are shown. (*C*) Length of the phases. (*D*) TE abundance at the beginning of the phase. (*E*) Abundance of cluster insertions at the beginning of the phase. (*F*) Stability of the phase. (*G*) Histogram showing the abundance of individuals with the given number of cluster insertions (at generation 1,000). ri, rapid invasion; sp, shotgun phase; ia, inactive.

piRNA clusters, even within a given species, may have profoundly different architectures. For example, in *Drosophila*, two specialized piRNA pathways exist which rely on different sets of piRNA clusters (Li et al. 2009; Malone et al. 2009). The somatic pathway mostly relies on a single cluster, that is *flamenco*, which is located in low recombining regions of the X-chromosome. The germline pathway, on the other hand, relies on several clusters (≈142) that are distributed over multiple chromosomes (Brennecke et al. 2007). We hypothesized that this difference in architecture may have an impact on invasion dynamics. To test this idea, we simulated five chromosomes with a size of 2 Mb, a piRNA cluster size of 1 Mb (i.e., 10% of the genome) and varied the number, the recombination rate and the genomic location of the clusters, while keeping the total size of piRNA clusters constant (fig. 4*A*). A single cluster in nonrecombining regions resembles the somatic architecture (flamenco-model) and multiple clusters distributed over five chromosomes resembles the germline architecture (germline-model; fig. 4*A*). For each architecture, we simulated 100 replicates. We found a pronounced difference of TE invasion dynamics between the flamenco- and germline-model (fig. 4*B*;supplementary table 3, Supplementary Material online). Although the length of the rapid invasion phase is significantly longer in the germline-model, the length of the shotgun phase is significantly longer in the flamenco-model (fig. 4*C*;supplementary table 3, Supplementary Material online). Notably, the number of TE insertions accumulating during an invasion is much lower in the flamenco-model than in the germline-model (fig. 4*D*;supplementary table 3, Supplementary Material online). Also, the number of cluster insertions necessary to stop an invasion is significantly lower with the flamenco-model (fig. 4*E*;supplementary table 3, Supplementary Material online). Finally, the stability of the shotgun phase is highest in the flamenco-model (Wilcox rank sum test, P<2.2e−16; fig. 4*F*;supplementary table 3, Supplementary Material online). This result raises the question what causes these pronounced differences between the flamenco- and the germline-model. We suggest that recombination, due to the random assortment of cluster insertions located on different chromosomes, is responsible. Recombination among cluster sites will generate individuals with multiple redundant cluster insertions but also individuals with few or no cluster insertions. The TE will be active in these individuals devoid of cluster insertions. Recombination thus leads to an inefficient silencing where on the average about four cluster insertions per diploid are necessary to furnish the majority of individuals with at least one cluster insertion. This hypothesis is in agreement with our results. Under the germline-model, individuals carry various numbers of cluster insertions, whereas in the flamenco-model the vast majority carries exactly two (fig. 4*G*). The few individuals with three (four) cluster insertions in the flamenco-model are likely due to multiple simultaneous insertions into the cluster at the same generation. To further test if recombination is responsible for the differences between the flamenco- and the germline-model, we simulated an additional architecture: a single trap with a recombination rate of 4 cM/Mb (i.e., flamenco-model with recombination; fig. 4*A*, setup 1). We found that the invasion dynamics of the flamenco-model with recombination are similar to the germline-model (fig. 4; supplementary table 3, Supplementary Material online), confirming the important role of recombination. In *Drosophila*, most germline clusters are located in heterochromatic regions which usually have a reduced recombination rate (Brennecke et al. 2007; Ellermeier et al. 2010). We asked whether the absence of recombination in germline clusters has an influence on the invasion dynamics. Therefore, we simulated an architecture where we allow recombination in the clusters that are distributed over the five chromosomes (i.e., germline-model with recombination; fig. 4*A*, setup 4). We however found that the invasion dynamics of the germline-model with recombination are very similar to the germline-model (fig. 4; supplementary table 3, Supplementary Material online). Thus, any recombination in addition to the random assortment of multiple clusters located on different chromosomes only has a minor influence on invasion dynamics. This result is in agreement with our previous finding that recombination rate has little influence on invasion dynamics (supplementary fig. 4, Supplementary Material online), as the simulated scenario allowed for random assortment among clusters. In terms of invasion dynamics, the absence of recombination in germline clusters does not confer a benefit to the host.

We conclude that the flamenco-model, that is, a single nonrecombining cluster, is the most efficient architecture for stopping TE invasions. It allows for the quickest and most stable silencing response which also minimizes the amount of TEs accumulating during an invasion. Any form of recombination within/among traps, either by random assortment of chromosomes or cross-overs, renders the silencing less efficient. Note that we solely evaluated the influence of the cluster architecture. Differences in size and insertion bias were not considered (see Discussion).

**Fig. 5.**
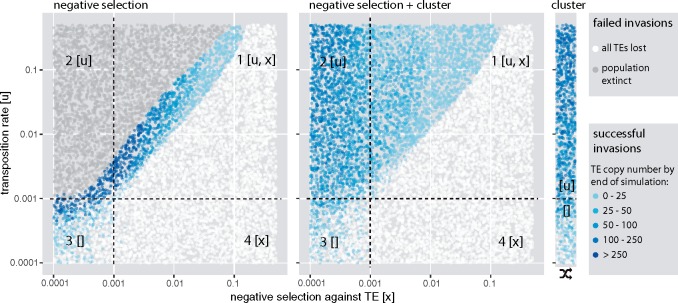
piRNA clusters protect populations from extinction due to an unchecked proliferation of deleterious TEs. Each dot represents the outcome of a single simulated TE invasion at generation 10,000. The transposition rate (*u*) and negative selection against TEs (*x*) were randomly picked. Results are shown for three different models where the following factors counteract the spread of TEs: (*A*) negative selection against TEs, (*B*) negative selection and piRNA clusters, and (*C*) solely clusters. Dependent on the efficacy of negative selection and transposition (N*u>1 and N*x>1 with *N *=* *1,000), the parameter space can be divided into four quadrants. Factors that are effective in a given quadrant are shown in brackets. Note that piRNA clusters greatly extend the parameter space over which TE invasions are feasible.

Classic works conducted before the discovery of the piRNA pathway showed that the accumulation of TEs could be stopped by negative selection against TEs (Charlesworth and Charlesworth 1983; Charlesworth and Langley 1989). In this work, we show that piRNA clusters may also stop TE invasions (fig. 1). It is feasible that piRNA clusters and negative selection against TEs jointly influence the dynamics of TE invasion. We therefore investigated the interaction of these two factors. Importantly, negative selection against TEs could readily remove all segregating TE insertions from a population. For the following simulations, we thus abrogated the previous requirement for successful invasions. Nevertheless, to avoid the stochastic early phase of invasions we initiated each simulation with 1,000 randomly distributed TE insertions (frequency of insertion f=1/2N). Initially, we simulated a model where all TEs, including cluster insertions, reduce the fitness of the host by an equal amount (w=1−xn, where *w* is the host fitness, *x* the negative effect of TEs, and *n* the TE copy number in an diploid individual). We explored the viable parameter space for TE invasions by randomly picking a negative effect (*x*) and a transposition rate (*u*). We than followed the resulting invasion up to 10,000 generations and recorded the result (fig. 5). Interestingly, in a model, where solely negative selection counteracts TEs (w=1−xn), successful invasions are only observed in a narrow parameter space (fig. 5). If negative selection is too strong all TE insertions will be lost. If the transposition rate is too high, negative selection cannot prevent the accumulation of TEs and the population will go extinct (average fitness drops to <0.1). To extend the viable parameter space, it was suggested that host fitness may not decrease linearly with TE copy numbers but exponentially instead ($w=1-xn^{t}$, where *t* is an exponential factor) (Charlesworth and Charlesworth 1983; Charlesworth and Langley 1989; Charlesworth 1991). It was reasoned that ectopic recombination between TEs could have a major impact on host fitness, and that the amount of ectopic recombination may exponentially increase with TE copy numbers (Charlesworth and Charlesworth 1983; Charlesworth and Langley 1989; Barrón et al. 2014). Although this exponential model extends the viable parameter space somewhat, populations still go extinct when transposition rates are high (supplementary fig. 5, Supplementary Material online). Interestingly, introducing piRNA clusters into the model (with w=1−xn), greatly extends the parameter space over which TE invasions are feasible (fig. 5). piRNA clusters thus prevent a rampant accumulation of TEs and rescue populations from extinction, even when negative selection against TEs is weak.

**Fig. 6.**
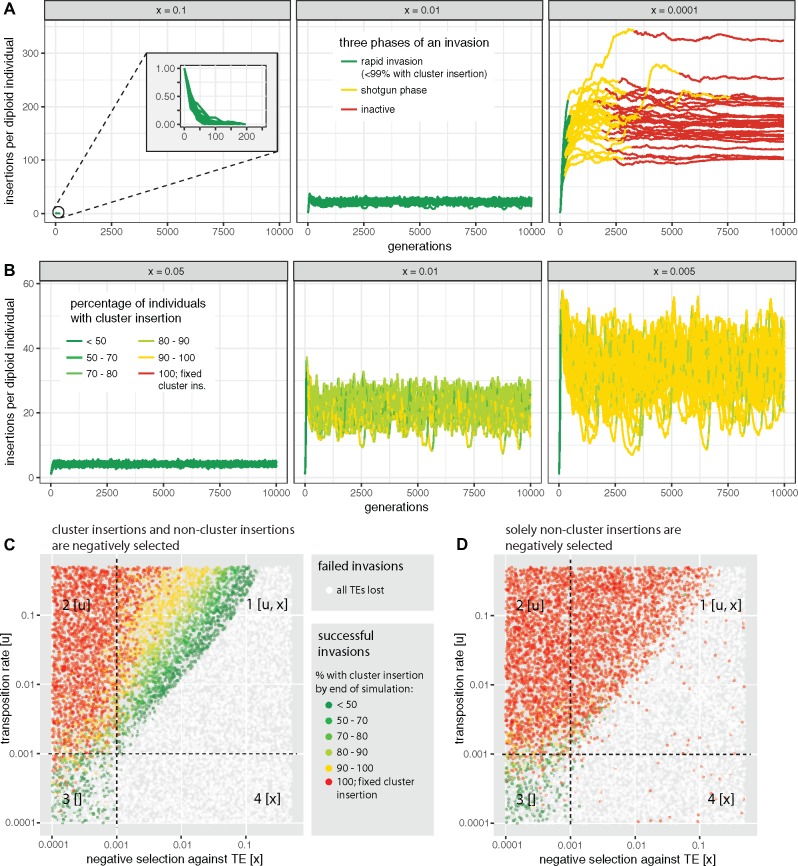
An equilibrium between transposition, negative selection against TEs and piRNA clusters (TSC balance) prevents fixation of TE insertions. TE copy numbers may thus stabilize although only some individuals in a population carry a cluster insertion. (*A*) Dependent on the strength of negative selection against TEs (*x*, top panel) an invasion may have three different principal outcomes: the TE may be lost (left panel), it may enter TSC balance (middle panel; these invasions never enter the shotgun phase), and the invasion may show the three typical phases (right panel). (*B*) During TSC balance, the fraction of individuals with cluster insertions depends on the strength of negative selection against TEs (*x*, top panel). Note that TSC balance prevents fixation of TE insertions, including cluster insertions and that negative selection influences TE abundance at the equilibrium. (*C*) Parameter space at which TSC balance may be observed (green and yellow dots in first quadrant). Each dot represents the outcome of a single simulated TE invasion at generation 10,000. Dependent on the efficacy of negative selection and transposition (N*u>1 and N*x>1 with *N *=* *1,000), the parameter space can be divided into four quadrants. Factors that are effective in a given quadrant are shown in brackets. (*D*) TSC balance is not observed when cluster insertions are neutral.

In a model where negative selection against TEs and piRNA clusters counteract the spread of TEs, three different outcomes are feasible (fig. 6*A*). In the case where negative selection against TEs is strong, all TE copies are quickly purged from the population (fig. 6*A*, left panel). If negative selection against TEs is weak the invasion has the three phases described before (fig. 6*A*, right panel). Interestingly, for intermediate levels of negative selection against TEs, TE copy numbers reach a stable plateau, although fewer than 99% of individuals carry a cluster insertion (fig. 6*A*, central panel). Furthermore, cluster insertions are not getting fixed and the TE will remain persistently active. We thus found a novel equilibrium state where both piRNA clusters and negative selection against TEs, counteract the spread of the TEs. In analogy to the classic transposition-selection balance (Barrón et al. 2014), we refer to this novel equilibrium state as “transposition-selection-cluster balance” (TSC balance). Next, we asked how many individuals actually carry cluster insertions during TSC balance. The fraction of individuals with cluster insertions depends on the strength of negative selection against TEs (fig. 6*B*; Kruskal–Wallis test at generation 10,000; $\chi^{2}=520.9$, df = 2, P<2.2e−16). When negative selection against TEs is strong only few individuals carry cluster insertions. Negative selection also influences the average number of TE insertions per individual, where fewer TEs are found when negative selection is strong (fig. 6*B*; Kruskal–Wallis test at generation 10,000, $\chi^{2}=472.01$, df = 2, P<2.2e−16). Next, we explored the parameter space at which TSC balance may occur (fig. 6*C*). Interestingly, TSC balance is mostly observed in the quadrant where both negative selection and transposition are effective (N*u>1 and N*x>1; fig. 6*C*, quadrant 1). According to basic population genetics theory a factor, such as negative selection against TEs (*x*), is only stronger than drift if the condition N*x>1 is met (Gillespie 2010). This observation confirms that TSC balance is a three component equilibrium, where negative selection and piRNA clusters jointly counteract the proliferation of TEs. If negative selection is weak solely piRNA clusters counteract the spread of the TE (fig. 6*C*, quadrant 2) and if negative selection is strong all TE copies will be removed from the population (fig. 6*C*, quadrant 4).

Finally, we asked if cluster insertions are positively selected during TSC balance. The total fitness effect of a cluster insertion is the sum of its direct (selection coefficient) and indirect effect, which results from the fact that cluster insertions repress TE activity and may thus be located on haplotypes that carry fewer deleterious TE insertions than haplotypes without cluster insertion. The overall sum of direct and indirect effect may be positive, even when the direct effect is negative as in this model. Identification of positive selection however requires comparing the allele frequencies of cluster insertions to insertions in neutral reference regions (“pseudo-small-RNA sites” in Kelleher et al. [2018]). Additional simulations with reference regions show that cluster insertions have significantly lower allele frequencies than reference insertions and are thus negatively selected (supplementary figs. 5 and 6 and table 4, Supplementary Material online). However, cluster insertions have higher allele frequencies than genomic insertions (noncluster and nonreference) and are thus less deleterious to host fitness than genomic insertions (supplementary fig. 6 and table 4, Supplementary Material online).

So far, we assumed that negative selection is equally acting against all TE insertions, including cluster insertions. However, it is feasible that cluster insertions incur no or only weak fitness costs. In this scenario, TSC balance is not observed (fig. 6*D*). Instead, cluster insertions are quickly fixed and the TE is inactivated in most of the cases (fig. 6*D*). Moreover, all invasions show the three characteristic phases described before (supplementary fig. 7, Supplementary Material online). Negative selection is again a major factor influencing TE abundance (supplementary fig. 7, Supplementary Material online; Kruskal–Wallis test at generation 10,000; $\chi^{2}=33.58$, df = 2, P=5.1e−8). We found that most noncluster insertions are eventually weeded out by negative selection under this model (supplementary fig. 8, Supplementary Material online). Hence, mostly cluster insertions persist within populations. This model thus predicts that piRNA clusters could contain insertions from families that are not found anywhere else in the genome. In agreement with this prediction, a careful annotation of the *flamenco* locus found insertions of families that are rare in *D. melanogaster* such as *Pifo* and *Phiddipo* (Zanni et al. 2013). Finally, we asked if cluster insertions are positively selected under this model. We performed additional simulations with reference regions included into genomes (supplementary fig. 5, Supplementary Material online). Cluster insertions have significantly higher allele frequencies than reference insertions (supplementary fig. 8 and table 5, Supplementary Material online). Hence, cluster insertions are positively selected under this model (see also Li et al. 2009; Kelleher et al. 2018).

In summary, we found that TE invasion may enter a novel equilibrium state, TSC balance, when two conditions are met: 1) all TEs including cluster insertions are negatively selected and 2) both negative selection and transposition are effective in the population. During TSC balance, TE copy numbers remain stable although only some individuals within a population carry a cluster insertions. Since cluster insertions are not getting fixed, the TE will remain persistently active.

**Fig. 7.**
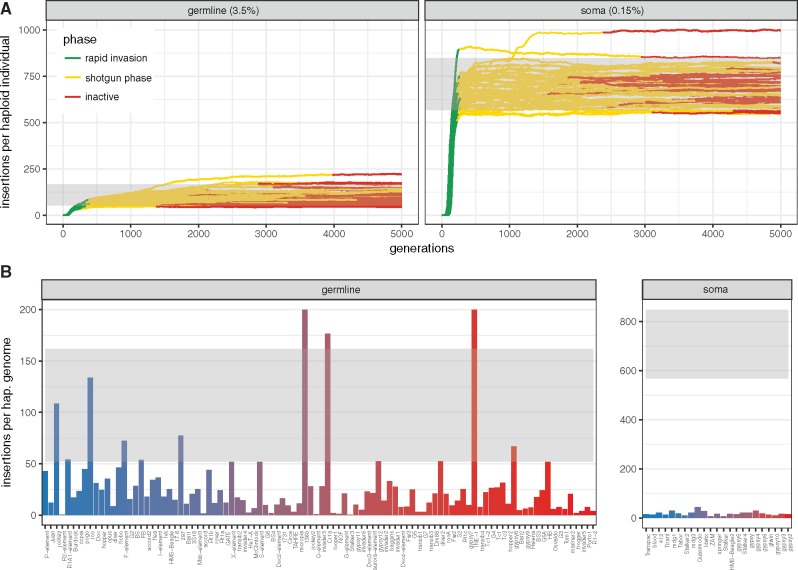
In *D. melanogaster*, the trap model roughly accounts for the abundance of germline TEs but fails to explain the abundance of somatic TEs. (*A*) Expected TE invasions for germline and somatic TEs in *D. melanogaster*. Simulated germline clusters are distributed over five chromosomes and account for 3.5% of the genome. A single nonrecombining cluster accounting for 0.15% of the genome (*flamenco*) was simulated for somatic TEs. Expected TE abundance between the 5% and 95% quantile is shown in gray shade. (*B*) Abundance of TE families in *D. melanogaster* compared with expectations. Gray shades indicate the expected TE abundance derived from the simulations (*A*). Color of bars indicates the average population frequency of a family (*blue* = 0.1, *red* = 1.0). Data are from Kofler, Nolte, et al. (2015).

We found that the number of TEs accumulating during an invasion is mostly influenced by the size and architecture of piRNA clusters. The transposition rate, the genome size, the recombination rate, the population size, and the excision rate solely had a minor influence on TE abundance. This finding is fortunate as it allows us to compute the expected TE abundance under the trap model for organisms with known cluster size and architecture, without having to rely on estimates for parameters that are hard to ascertain, such as the transposition rate. Comparing the expected and the observed TE abundance will allow to test whether the trap model holds for an organism of interest. *Drosophila melanogaster* is ideally suited for this analysis as both the architecture of piRNA clusters as well as the TE abundance are known (Brennecke et al. 2007; Kofler, Nolte, et al. 2015). We first computed the expected TE abundance for germline and somatic TEs (fig. 7*A*). In the simulations, we assumed that germline clusters are distributed over five chromosomes and account for 3.5% of the genome, whereas the sole somatic cluster (e.g., *flamenco*) accounts for 0.15% of the genome (assuming a *flamenco* size of 300 kb and a genome size of 200 Mb, Brennecke personal communication [Bosco et al. 2007]). According to these simulations, germline TEs in *D. melanogaster* should have about 52–162 insertions per haploid genome, whereas somatic TEs should have about 568–848 insertions (90% confidence interval; fig. 7*A*). When comparing these expectations to the TE abundance observed in a natural population from South Africa (Kofler, Nolte, et al. 2015) we found that the abundance of germline TEs fits the prediction reasonably well (fig. 7*B*). The TE abundance is slightly lower than expected which could be due to negative selection against TEs (simulations are based on a neutral model). However, the abundance of somatic TEs is substantially lower than expected (fig. 7*B*). This estimate is even conservative as our simulations did not consider that cluster insertions in *flamenco* need to be antisense (effectively doubling the expectations for *flamenco*). Also, hitherto undetected recombination within *flamenco* cannot explain the discrepancy, as recombination would lead to increased expectations for somatic TEs, thus exacerbating the problem. We thus conclude that the trap model does not account for the abundance of somatic TEs. What could be responsible for this pronounced discrepancy? It is feasible that some somatic TEs have an insertion bias into the *flamenco* locus. For example, the somatic TE *gypsy* has a chromodomain that interacts with repressive heterochromatin, which allows targeting heterochromatic regions where also many piRNA clusters are found (Sultana et al. 2017). Alternatively, it is possible that in addition to piRNAs also siRNA act to repress TEs in the soma (Barckmann et al. 2018). The siRNA-based defense may be independent of cluster insertions and thus allow for a rapid silencing of invading TEs in the soma. Finally, it is feasible that somatic TEs are more deleterious than germline TEs. The virus like particles of somatic TEs that infect the germline could, for example, have deleterious consequences for development.

## Discussion

In this work, we explored the dynamics of TE invasions with piRNA clusters using individual based forward simulations. We assumed that a TE is active until a member of the family jumps into a piRNA cluster, whereupon all members of the family are inactivated. This view is known as the trap model. The trap model was initially suggested by Bergman et al. (2006), even before the discovery of piRNAs, as a means to provide hosts with an adaptive immunity against TEs. Bergman et al. (2006) suggested that once a TE jumps into a cluster of nested TEs a cosuppression network is activated which silences all members of the family. One year later this hypothesis received substantial support by the discovery of piRNAs, that is small RNAs that mediate the transcriptional and posttranscriptional silencing of TEs (Brennecke et al. 2007; Gunawardane et al. 2007; Sienski et al. 2012; Le Thomas et al. 2013). Based on the observations that piRNAs suppress TEs and that piRNAs are mostly produced from piRNA clusters, it was suggested that a TE jumping into a piRNA cluster triggers production of piRNAs complimentary to the TE, which then silence the TE (Malone and Hannon 2009; Zanni et al. 2013; Goriaux et al. 2014; Yamanaka et al. 2014; Ozata et al. 2019). This view is further supported by the finding that insertion of an artificial sequence into piRNA clusters results in piRNAs complimentary to the artificial sequence (Muerdter et al. 2012) and that piRNA clusters mostly consist of TEs (Brennecke et al. 2007; Malone et al. 2009; Zanni et al. 2013). Hence, piRNA clusters may contain the trapped remnants of past invasions. Direct support for the trap model comes from a study which found that a single *P-element* insertion in *X-TAS* (a piRNA cluster) is sufficient to silence all *P-element* copies in trans (Josse et al. 2007). It is however not clear if this observation holds for all transposons and piRNA clusters. It is conceivable that for some TEs more than one cluster insertion is necessary to suppress activity. Small RNA biology is a dynamic research field and it can thus not be precluded that future discoveries will necessitate a modulation of the trap model.

Initially, we explored invasion dynamics assuming neutral TE insertions and only later considered negatively selected TE insertions. This approach was chosen for two reasons. First, to dissect the behavior of a complex system it is important to start with a simple model and to extend the complexity of the model only gradually by taking additional influencing factors into account (Otto and Day 2007). Second, the fitness effects of TE insertions remain controversial (Arkhipova 2018). It seems unlikely that a sophisticated host defense against TEs, that is the piRNA pathway, would have evolved unless TEs have a negative fitness effect. This is in agreement with some previous studies that identified deleterious effects of TE insertions (Yukuhiro et al. 1985; Mackay 1989; Mackay et al. 1991; Houle and Nuzhdin 2004; Blumenstiel et al. 2014). Other studies however obtained more ambiguous results. If TE insertions have a direct negative effect, for example, by disrupting genes or promoter regions, and assuming that these mutation are recessive (e.g., if disrupted genes are haplosufficient) we expect fewer TEs in the X-chromosome than in autosomes, since the negative effect of X-linked TEs is directly exposed to selection in hemizygous males. However, in *Drosophila*, the X-chromosome has a similar TE density than autosomes, which argues against a strong direct effect of TE insertions (Petrov et al. 2011; Kofler et al. 2012). Negative fitness effects of TEs may also arise from ectopic recombination among elements at different sites, which may lead to highly deleterious genomic rearrangements (Montgomery et al. 1987; Langley et al. 1988). As a consequence, we expect a negative correlation between the recombination rate and the TE density (assuming that rates of ectopic and meiotic recombination are correlated). Although this correlation was found for *Drosophila*, it was not found for other organisms, such as *Caenorhabditis* and *Arabidopsis* (Quadrana et al. 2016; Kent et al. 2017; Laricchia et al. 2017). These findings led to some doubts about the importance of ectopic recombination in containing the spread of TEs (Quadrana et al. 2016; Kent et al. 2017). For these and other reasons, Arkhipova (2018) argues that neutrality should be the null hypothesis for any evolutionary studies of TEs. Using a neutral model, we found that a TE invasion consists of three distinct phases and identified factors that influence key properties of the phases. In our model, fixation of a cluster insertion permanently deactivates the TE. However, it is entirely possible that mutations within the sequence of a TE may enable the TE to escape from deactivation by piRNAs, thus triggering a novel wave of a TE invasion. If the sequences of piRNA clusters also evolve, an arms race between TEs and clusters may result, which may be interesting to explore in future theoretical works.

We also show that the population size is the major factor influencing the length of the unstable shotgun phase. Cluster insertions should thus segregate for extended periods of time in large populations. Fixation of a cluster insertion roughly requires 2*Ne generations (fig. 3*D*). Hence, in *D. melanogaster* with an estimated population size $>10^{6}$, TEs that invaded recently, like many LTR families, ought to have segregating cluster insertions (shotgun phase) (Kreitman 1983; Bowen and McDonald 2001; Bergman and Bensasson 2007). Older families, like many non-LTRs, on the other hand should mostly have fixed cluster insertions (inactive phase) (Bergman and Bensasson 2007). The phase of a family may also affect the activity. In case, cluster insertions are segregating (shotgun phase), the TE may still be active in a few individuals which randomly end up without any cluster insertion. Segregating cluster insertions may therefore account for the low level of activity observed for many TE families in *Drosophila* (e.g., transposition rate $u\approx 10^{-5}$ [Nuzhdin 1999]). Families with segregating cluster insertions may thus have a higher activity than families with a fixed cluster insertions.

Later, we introduced negative selection against TEs into our model. We assumed that all TE insertions, irrespective of the insertion site, have an equal contribution to host fitness. Although widely used in theoretical models of TE dynamics (e.g., Charlesworth and Charlesworth 1983; Marshall 2008b), this assumption may not hold when TEs have a direct negative effect, but the assumption may hold when ectopic recombination is responsible for the negative effect of TEs. However, we found that negative selection reduces the amount of TEs accumulating during an invasion (fig. 6*B*); see also Kelleher et al. (2018). More surprisingly we found that piRNA clusters dramatically extend the parameter space over which TE invasions are feasible. piRNA clusters prevent extinction of populations from an uncontrollable proliferation of TEs. This result is in agreement with the finding that piRNA clusters lower the fitness costs of TE insertions (Li et al. 2009). In our simulations, piRNA clusters account for 3% of the genome. It is feasible that smaller piRNA clusters may not be able to prevent extinction of populations over the entire parameter space. Surprisingly, we found that negative selection can have a dramatic effect on invasion dynamics. A TE invasion may enter a stable equilibrium, the TSC balance, where piRNA clusters and negative selection against TEs counteract the proliferation of a TE. TSC balance may be imagined as a form of balancing selection, not on a particular allele, but on the fraction of individuals with piRNA clusters. If few individuals have a cluster insertion, the TE will be highly active and novel cluster insertions will be generated. Thus, the number of individuals with cluster insertions increases. If most individuals have a cluster insertion the TE will be largely inactive and negative selection will weed out TE insertions, including cluster insertions. TSC balance would be deleterious to natural populations. Because cluster insertions are thwarted from fixation, the TE will remain persistently active. Novel TE insertions will thus generate a continuous load of deleterious TE insertions in a population. These considerations raise the question whether some families in natural populations are actually in TSC balance and how such a balance could be identified? Our simulations show that during TSC balance only some individuals in a population will carry cluster insertions for active TE families. This prediction could be tested by determining the abundance of cluster insertions for different families in individuals of natural populations. However, an important requirement for TSC balance is that cluster insertions are negatively selected, which could arise due to ectopic recombination between TEs or due to piRNA clusters bearing some cost to the host (e.g., metabolic cost of generating large quantities of piRNAs).

It has been argued that TE invasions may be stopped by hard sweeps of cluster insertions, that is, a single insertion in a piRNA cluster may be positively selected and rapidly rises in frequency (Blumenstiel 2011; Yamanaka et al. 2014). In this work, we suggest an alternative explanation: TE invasions are initially stopped by many segregating insertions in piRNA clusters (see also Kelleher et al. [2018]). This hypothesis is in agreement with our previous work where we had the opportunity to monitor a natural *P-element* invasion in experimentally evolving populations of *Drosophila simulans* (Kofler et al. 2018). The invasion plateaued around 20 generations at which time also the first *P-element* insertions in piRNA clusters were observed. In agreement with our model, all observed cluster insertions were segregating at low frequency (Kofler et al. 2018). However, we found cluster insertions solely for 15% of the investigated haploid genomes, whereas our neutral simulations predict that two cluster insertions per haploid genome are necessary to stop an invasion. It is possible that we missed several cluster insertions due to the incomplete *Drosophila simulans* assembly or that euchromatic *P-element* insertions have been converted into piRNA producing loci by paramutations (de Vanssay et al. 2012; Le Thomas et al. 2014; Mohn et al. 2014; Kofler et al. 2018). This work however raises a third possibility. The *P-element* invasion may have entered TSC balance. In this equilibrium state, it is not expected that all individuals carry piRNA producing *P-element* insertions. This prediction could be tested by sequencing the small RNAs of several individuals from a recently invaded population. Stable *P-element* copy numbers in the absence of piRNAs against the *P-element* in some individuals would support TSC balance.

Our simulations of TE invasions highlighted areas that need more attention and offers several hypothesis that could be tested. Most importantly, our work showed the profound impact of negative selection against TEs on the dynamics of TE invasions. It will thus be crucial to obtain reliable estimates of the distribution of fitness effects for TE insertions, ideally for cluster insertions and noncluster insertions separately. Furthermore, it will be important to test if all TE families could be repressed by a single cluster insertion. The shotgun silencing model predicts that recently active TE families, such as most LTRs in *D. melanogaster*, should have segregating cluster insertions. Moreover, per diploid we expect on the average two insertions in somatic clusters and 3.8 in germline clusters. These hypotheses can be tested by assembling and annotating piRNA clusters for multiple individuals of a population. Given the progress of long read sequencing and scaffolding techniques such as Hi-C, this aim has come within reach (Dudchenko et al. 2017; Kuderna et al. 2019). This work also raises the possibility that some TEs could be in TSC balance. This hypothesis can be tested by estimating the piRNA content for several individuals of a natural population. TSC balance could be refuted for a given family if all individuals in the population have piRNAs complimentary to the family. Finally, it will be important to determine why the trap model does not hold for somatic TEs. Monitoring an experimental invasion of a somatic TE at the genomic level could provide insights.

## Materials and Methods

### Simulations

To simulate the dynamics of TE invasion, we developed “Invade,” a novel Java tool that performs individual based forward simulations of TE invasions under different models. This tool builds on Java libraries developed for previous works (Kofler, Nolte, et al. 2015; Vlachos and Kofler 2018). Invade allows to specify a wide range of different parameters such as the genomic architecture (number and size of chromosomes), the recombination rate, the architecture of piRNA clusters, the population size, the transposition rate, the excision rate, negative selection against TEs, and the TE abundance in the starting population. The tool also provides diverse summary statistics as output, such as the site frequency spectrum of TEs and the TE abundance in individuals of a population. At each generation Invade performs the following steps in the given order 1) mate pairs are formed based on the fitness of the individuals, 2) haploid gametes are generated based on the recombination map, 3) TE excisions are introduced, 4) novel TE insertions are introduced, 5) zygotes are formed, 6) piRNA cluster insertions are counted, 7) the fitness of the individuals is computed, and 8) the output is generated (optional). To minimize the parameter space, we performed simulations with default conditions and varied solely the parameter of interest. Per default, we used a genome consisting of five chromosomes with size 10 Mb (*–genome mb: 10, 10, 10, 10, 10*), a recombination rate of 4 cM/Mb (*–rr cm_mb: 4, 4, 4, 4, 4*), a piRNA cluster at the beginning of each chromosome with a total size of 3% of the genome (*–cluster kb: 300, 300, 300, 300, 300*), a transposition rate of 0.1 (*–u 0.1*), an excision rate of zero (*–v 0.0*), neutral TE insertions (*–x 0.0*), a population size of 1,000 (*–N 1000*), and 10 TE insertions randomly distributed in the starting population (*–basepop seg: 10*).

To test if cluster insertions are selected, we included neutral reference regions into the simulations. We developed a novel branch of Invade for this task (Invade_bps.jar). Ideally the reference regions should not (or as little as possible) interfere with invasion dynamics. Therefore, TEs inserted into reference regions do not repress TE activity, do not transpose and have no direct effect on host fitness (similarly to pseudo-small-RNA sites in Kelleher et al. [2018]). We simulated reference regions that mirrored the architecture of piRNA clusters but were located on the opposite ends of chromosomes (supplementary fig. 5, Supplementary Material online; *–ref-sites kb: 300, 300, 300, 300, 300*).

### Data Analysis

Data were analyzed using custom Python scripts which are available as part of the te-tools package (https://sourceforge.net/projects/te-tools/; all scripts used in this work are in the folder sim3p). This package includes scripts for annotating the phases of the TE invasions (*phasing.py*) and computing summary statistics for the phases, such as the length of a phase and the TE abundance at the beginning of a phase (*abundance-of-phase.py*, *variance-of-phases.py*, *cluinsabundance-of-phase.py*, *length-of-phases.py*). Statistical analysis was performed in R (R Core Team 2012) and visualization was done with ggplot2 (Wickham 2016).

### Details on Simulated Scenarios

Differences in cluster size were simulated by scaling the size of all clusters proportionally. For example, to obtain clusters that account for 30% of the genome we simulated piRNA clusters with a size of 3,000 kb (*–cluster kb: 3000, 3000, 3000, 3000, 3000*). To simulate differences in genome size we scaled the size of each chromosome and cluster proportionally. For example, to simulate a genome of size 500 Mb we used five chromosomes of size 100 Mb and five clusters of size 3 Mb (*–genome mb: 100, 100, 100, 100, 100 –cluster kb: 3000, 3000, 3000, 3000, 3000*). Note that this approach maintains the genomic proportion of clusters at the default value of 3%. When evaluating the impact of excision rate we kept the net transposition rate (u′=u−v; i.e., transpositions minus excisions) at the default value of u′=0.1. For example, to simulate 10% excisions, we used a transposition rate of *u *=* *0.111111 and an excision rate of *v *=* *0.0111111. With an excision rate of 0% the net transposition rate is identical to the transposition rate (u′=u).

## Availability

Invade is implemented in Java and distributed under the GPLv3 at https://sourceforge.net/projects/invade/; last accessed April 18, 2019.

## Supplementary Material

Supplementary data are available at *Molecular Biology and Evolution* online.

## Supplementary Material

Supplement_Material_msz079
