# Supplementary material for: Dynamics of Transposable Element Invasions with piRNA Clusters
Source: Mol Biol Evol. 2019 Apr 9;36(7):1457–72. doi: 10.1093/molbev/msz079 (PMC6573471; doi:10.1093/molbev/msz079)
Supplement: Supplement_Material_msz079 [file supplement_material_msz079.pdf]

Supplement to "Dynamics of transposable element invasions with piRNA clusters"

Robert Kofler

February 15, 2019

Supplementary figures

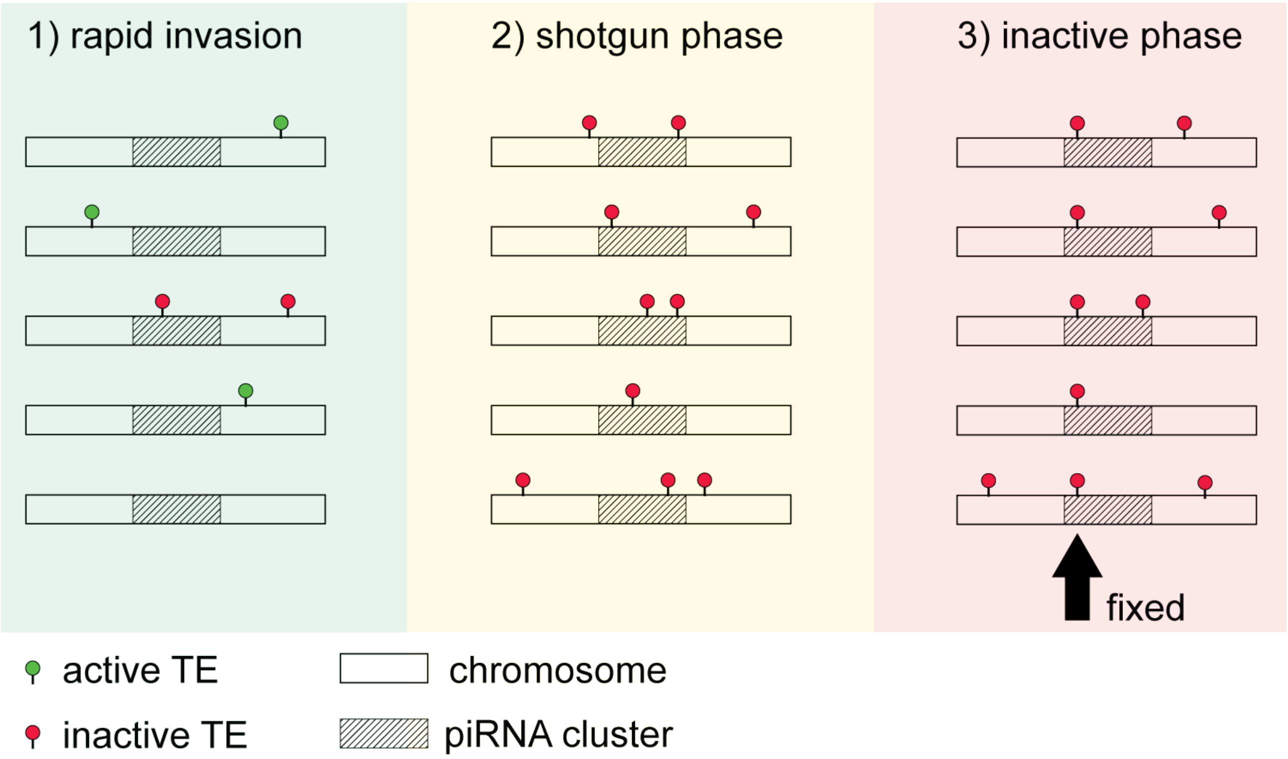

Supplementary Figure 1: Overview of the three phases of a TE invasion. For simplicity we show a population of five haploid individuals for each phase. At the "rapid invasion" phase the TE is active and spreads in a population. Some individuals may have already acquired a cluster insertion. At the "shotgun phase" the TE is silenced by segregating cluster insertions. More than 99% of the individuals carry at least one cluster insertion. At the "inactive phase" the TE is persistently inactivated by at least one fixed cluster insertions. Additional cluster insertions may however still be segregating.

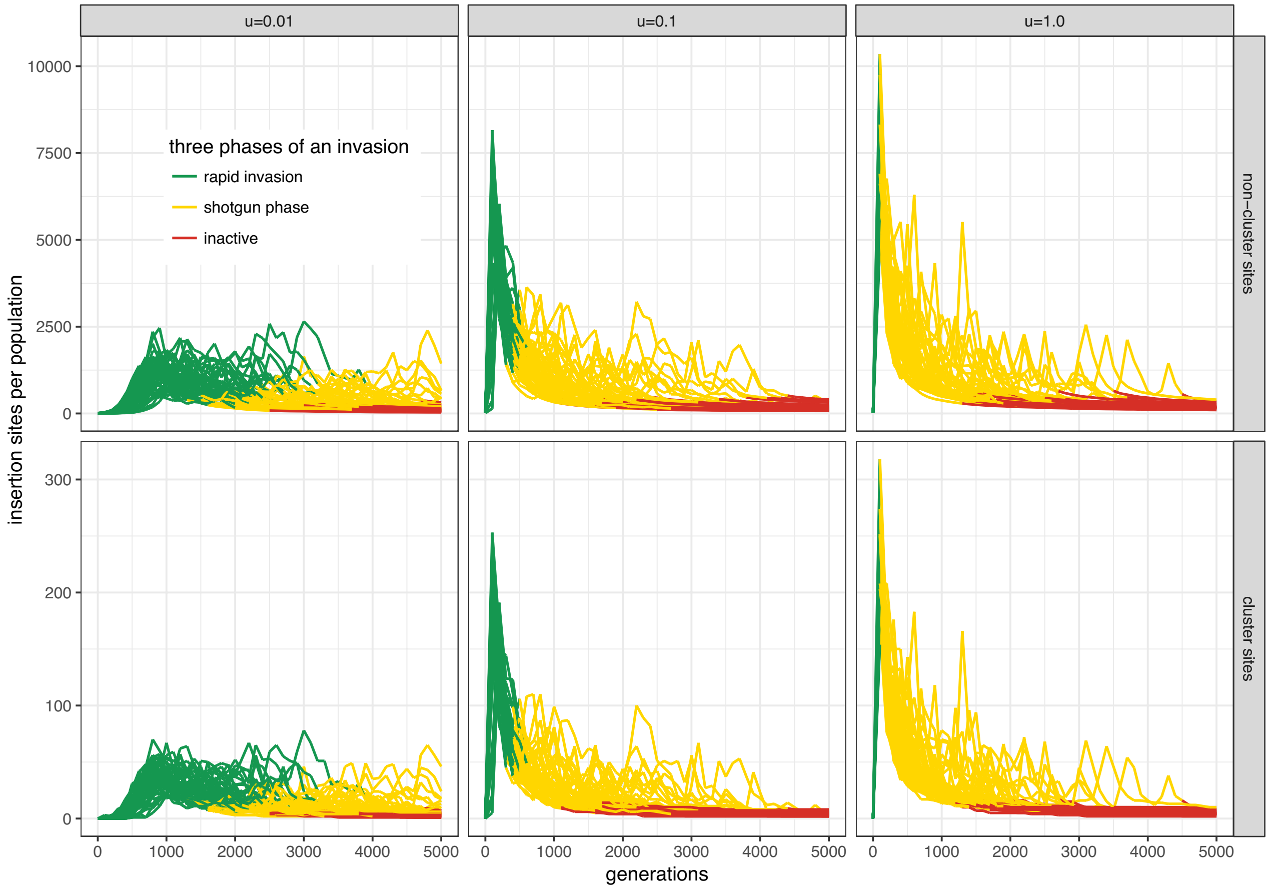

Supplementary Figure 2: Insertion sites in a population during a TE invasion. Fifty replicates are shown for each transposition rate (u, top panel). Results are shown separately for cluster insertions and non-cluster insertions (right panel).

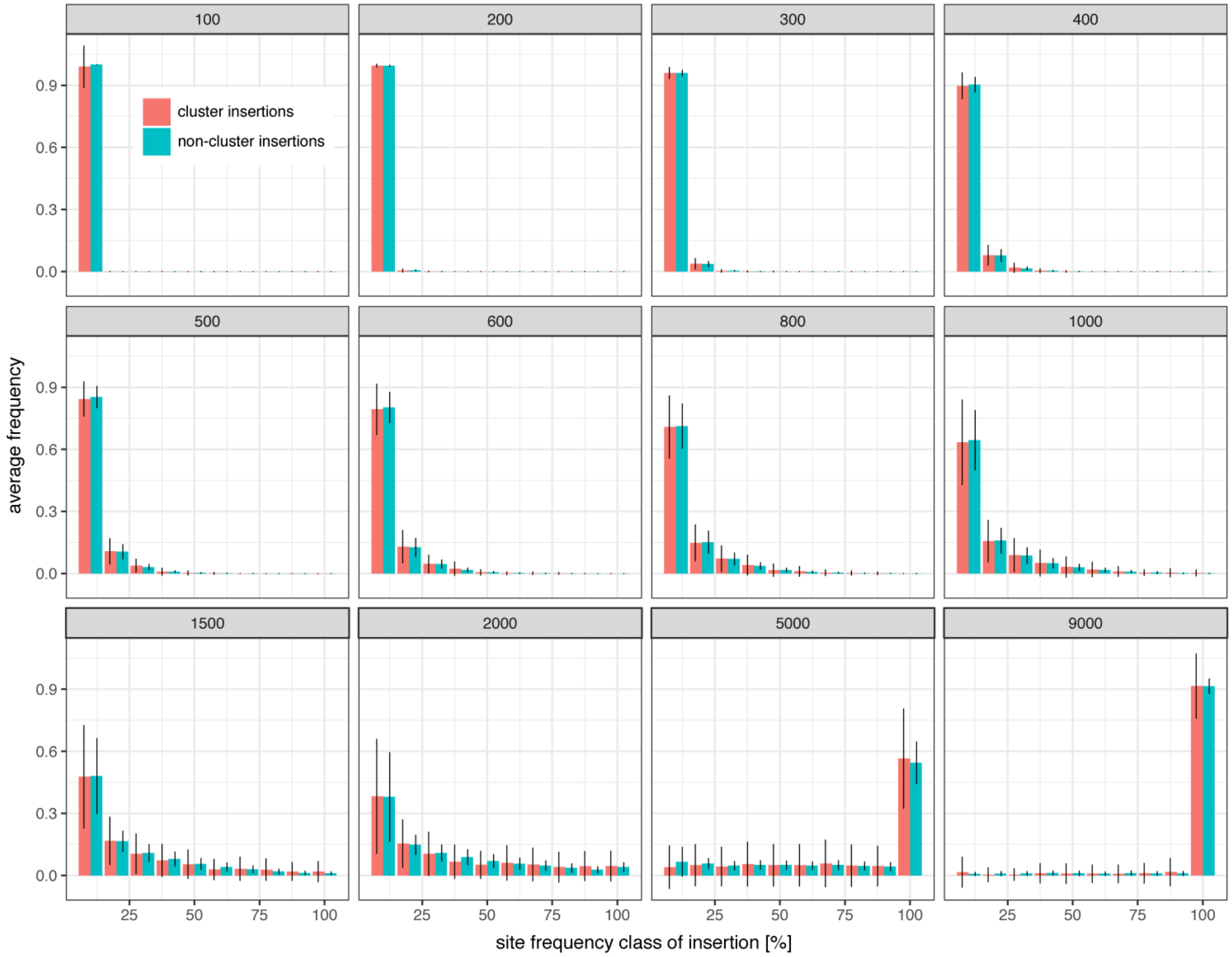

Supplementary Figure 3: Site frequency spectrum of cluster and non-cluster insertions during a TE invasion. Neutral TE insertions were simulated. The generation is shown in the top panel and error bars are based on 100 replicates.

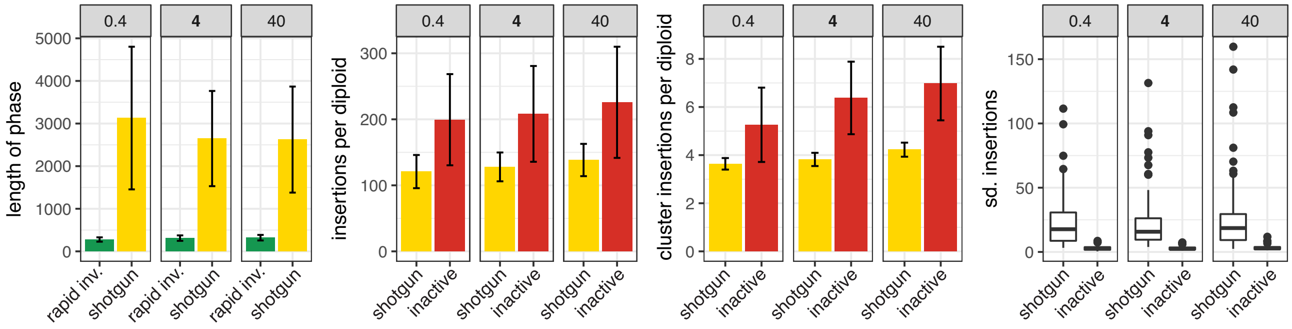

Supplementary Figure 4: Influence of the recombination rate on TE invasions. We simulated 100 replicates for each recombination rate (top panel; in cM/Mb). The default recombination rate is shown bold.

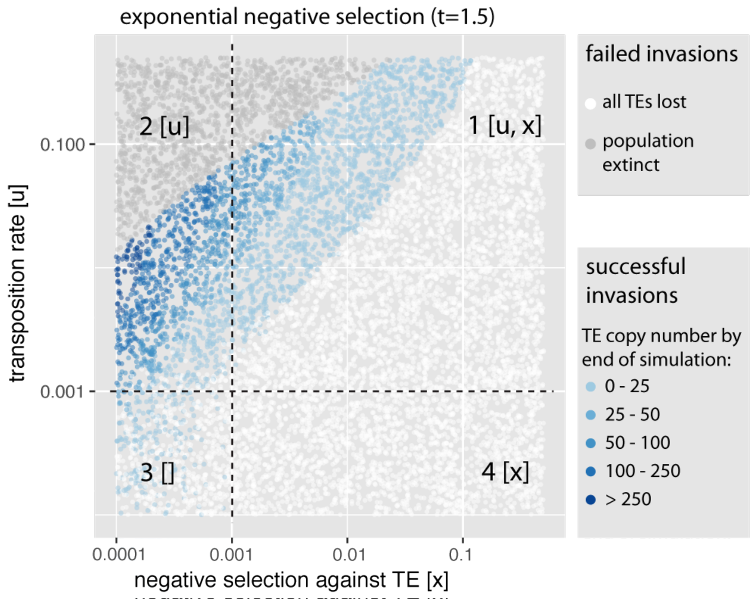

Supplementary Figure 5: Parameter space over which successful TE invasion are feasible when negative selection against TEs increases exponentially. Following Charlesworth and Charlesworth [1983] we used the equation $w = 1 - xn^t$ to calculate the host fitness, where x is the negative selection against TEs, n the TE copy number and t an exponential factor that we set to $t = 1.5$. Each dot represents the outcome of a single simulated TE invasion at generation 10.000. The transposition rate (u) and the negative selection against TEs (x) were randomly drawn. Dependent on the efficacy of negative selection and transposition ($N * u > 1$ and $N * x > 1$ with $N = 1000$) the parameter space can be divided into four quadrants. Factors that are effective in a given quadrant are shown in brackets.

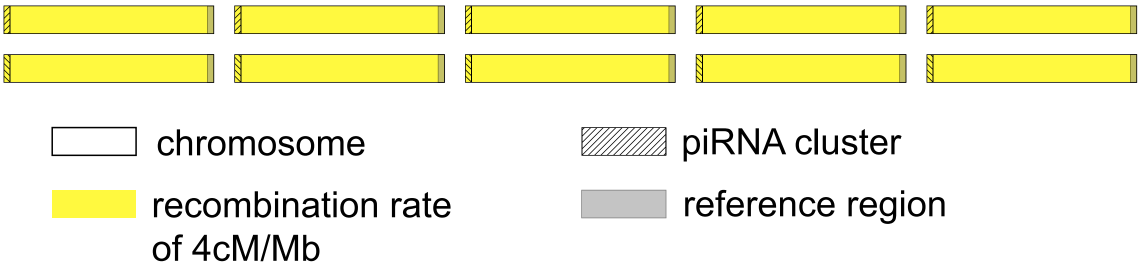

Supplementary Figure 6: Genomic architecture for simulations with reference sites. We simulated 5 chromosomes of size 10Mb for a diploid organism. Each chromosome carried a piRNA cluster of size 300kb at one end and a reference region of size 300kb at the opposite end. A constant recombination rate of 4 cM/Mbp (yellow) was used.

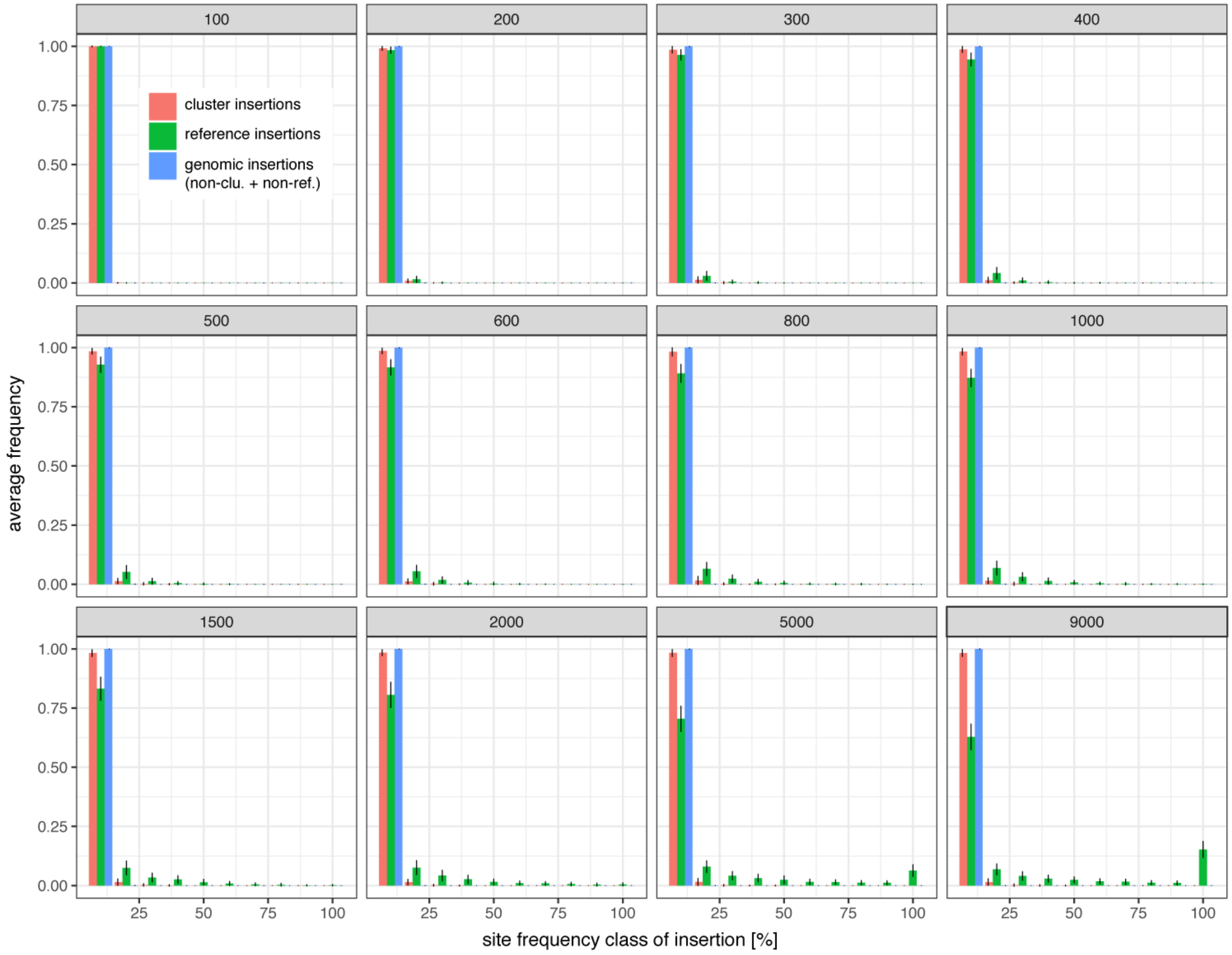

Supplementary Figure 7: Site frequency spectrum of cluster, reference and genomic (i.e. non-cluster and non-reference) insertions during a TE invasion. An identical negative effect on host fitness ($x = 0.01$) was simulated for genomic and cluster insertions. Reference insertions were neutral. The generation is shown in the top panel and error bars are based on 100 replicates.

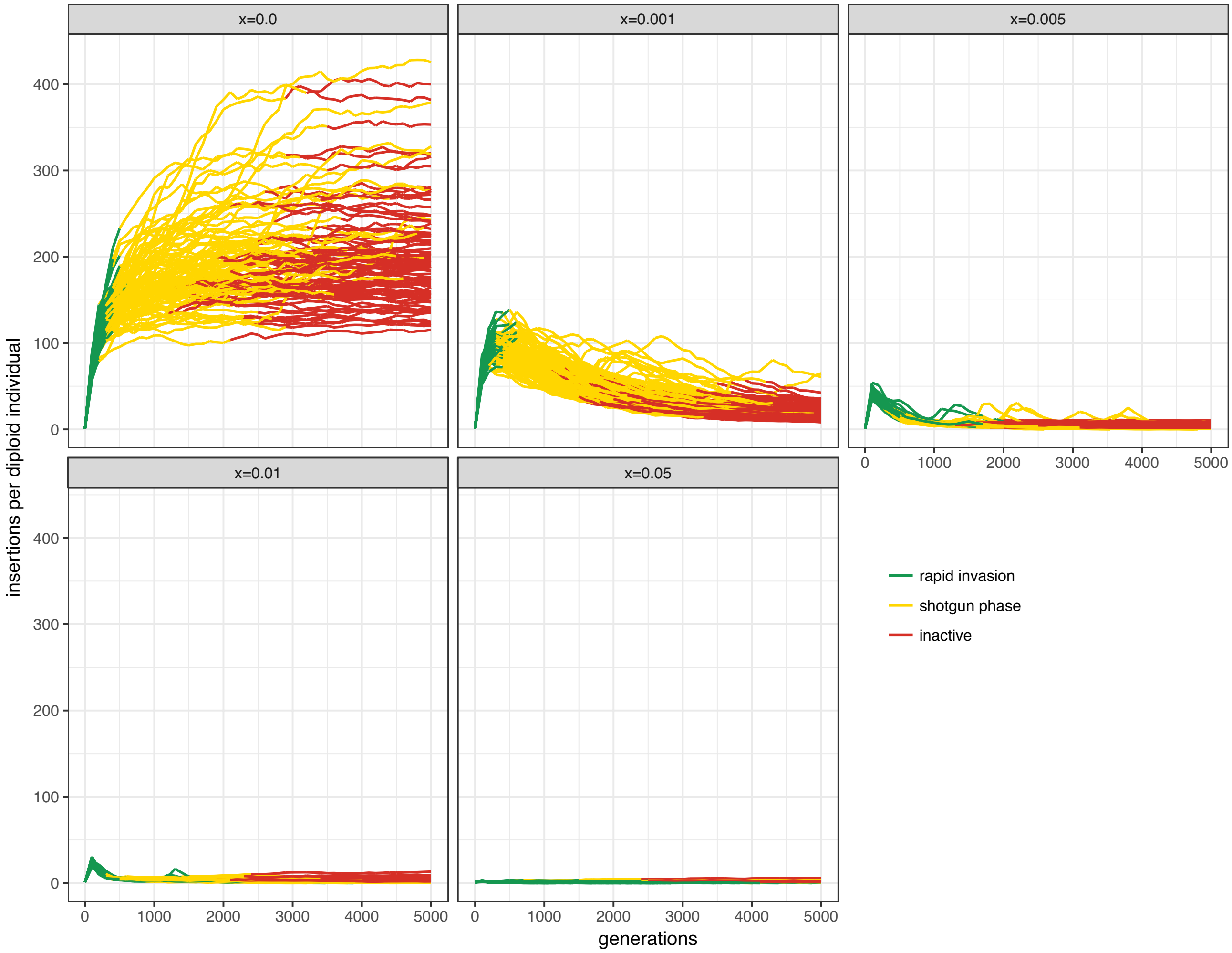

Supplementary Figure 8: Abundance of TE insertions per diploid individual during a TE invasion. We used a model where non-cluster insertions are negatively selected and cluster insertions are neutral. Fifty replicates are shown for each simulated selection coefficient (x; top panel). Note that $x = 0.0$ corresponds to a model where cluster and non-cluster insertions are neutral.

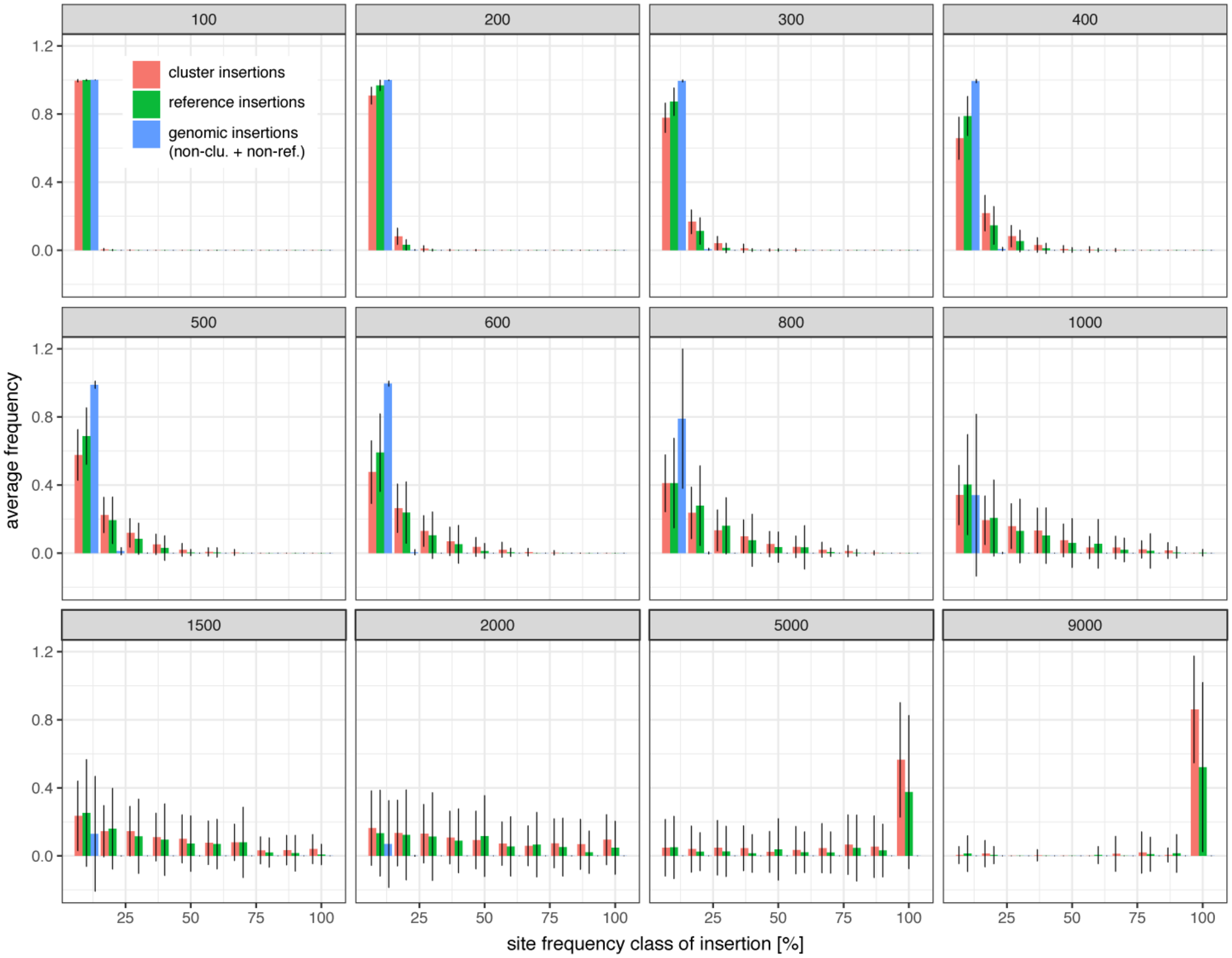

Supplementary Figure 9: Site frequency spectrum of cluster, reference and genomic (i.e. non-cluster and non-reference) insertions during a TE invasion. Only genomic insertions were simulated to have a negative impact on the fitness of the host ($x = 0.01$). Reference and cluster insertions were simulated neutral ($x = 0.0$). The generation is shown in the top panel and error bars are based on 100 replicates. The higher allele frequency of cluster insertions relative to reference insertions suggest that cluster insertions are positively selected. Note that genomic insertions were lost in most replicates by generation 5000.

Supplementary tables

Supplementary Table 1: Average population frequency (f) of cluster (c) and non-cluster (nc) during a TE invasion. We simulated neutral TE insertions. The significance of the allele frequency difference across 100 replicates was computed with a Wilcoxon rank sum tests. Apart from the last generation the allele frequency between cluster and non-cluster insertions is not significantly different. gen. generation

gen.	f_c	f_{nc}	$p_{c \leftrightarrow nc}$
100	0.003	0.002	0.787
200	0.012	0.011	0.949
300	0.022	0.022	0.867
400	0.036	0.035	0.755
500	0.047	0.045	0.846
600	0.060	0.057	0.590
800	0.087	0.084	0.784
1000	0.117	0.110	0.742
1500	0.204	0.192	0.931
2000	0.285	0.271	0.573
5000	0.763	0.733	0.075
9000	0.955	0.954	7.53e-11

Supplementary Table 2: Impact of different factors on key properties of TE invasions. We evaluated the influence of the transposition rate (u), the genome size (gs), the cluster size (cs), the population size (N), the excision rate (v) and of the recombination rate (rr). To quantify the impact of the factors we computed the ratio between the maximum and the minimum value for each property (e.g. the ratio between the maximum and minimum length of the phase). A ratio around 1.0 indicates that the factor has little influence on the invasion, while a ratio $\gg 1$ indicates a large effect. Strong influences (> 2) are shown in bold. The significance of the effect was assessed with a Kruskal-Wallis rank sum test: * 0.05, ** 0.01, *** 0.001; phases: ri rapid invasion, sp shotgun phase, ia inactive

		u	gs	cs	N	v	rr
length of phase	ri	**64.7*****	1.15***	1.39***	**2.11*****	1.04	1.15***
	sp	1.12	1.14	1.13	**70.04*****	1.08	1.19
TE abundance	sp	1.21***	1.16***	**81.45*****	1.32***	1.92***	1.14***
	ia	1.88***	1.27***	**70.68*****	**2.01*****	1.86***	1.13*
trap abundance	sp	1.24***	1.14***	1.13***	1.38***	1.02^{+}	1.16***
	ia	1.85***	1.40***	1.12	1.90***	1.09	1.32***
stability	sp	**2.40*****	1.60***	**38.48*****	**2.33*****	1.89***	1.11
	ia	1.57***	1.12**	**8.06*****	**5.05*****	1.39***	1.12

Supplementary Table 3: Similarities between different cluster architectures. We computed the ratio between the architecture of interest and the germline-model (setup3) for multiple key properties of TE invasions. For example, the first value 0.63 states that the length of the rapid invasion phase of "setup2" is 63% of the length of the same phase in "setup3". A value of ≈1.0 implies that two architectures have a similar key property. Significance of the differences was computed with a Wilcoxon rank sum test: * 0.05, ** 0.01, *** 0.001; phases: ri rapid invasion, sp shotgun phase, ia inactive

property	phase	setup2/setup3[1]	setup1/setup3[2]	setup4/setup3[3]
length of phase	ri	0.63***	0.99	1.04
	sp	1.43***	1.08	0.91
TE abundance	sp	0.52***	0.78***	1.05*
	ia	0.39***	1.01	1.13*
trap abundance	sp	0.52***	0.77***	1.05***
	ia	0.39***	1.08	1.20***
stability	sp	0.34***	1.53***	1.23
	ia	0.47***	1.17*	1.21

[1] flamenco model vs. germline model
[2] flamenco with recombination vs. germline model
[3] germline with recombination vs. germline model

Supplementary Table 4: Average population frequency (f) of cluster (c), reference (r) and genomic (g; i.e. non-cluster and non-reference) insertions during a TE invasion. An identical negative effect on host fitness ($x = 0.01$) was simulated for genomic and cluster insertions. Reference insertions were neutral. The significance of the allele frequency difference across 100 replicates was computed with a Wilcoxon rank sum tests. Note that cluster insertions have a higher frequency than genomic insertions but a lower frequency than reference insertions. Under this model cluster insertions are therefore negatively selected, but they are less deleterious than genomic insertions. gen. generation

gen.	f_c	f_r	f_g	$p_{c \leftrightarrow r}$	$p_{c \leftrightarrow g}$
100	0.007	0.006	0.004	0.593	2.28e-32
200	0.012	0.014	0.005	7.42e-10	3.46e-34
300	0.012	0.018	0.005	6.11e-21	3.62e-34
400	0.012	0.022	0.005	1.32e-31	3.82e-34
500	0.012	0.026	0.005	9.75e-32	4.44e-34
600	0.011	0.029	0.005	7.48e-34	2.94e-34
800	0.012	0.037	0.005	4.93e-34	3.18e-34
1000	0.011	0.042	0.005	2.54e-34	2.58e-34
1500	0.012	0.060	0.005	2.55e-34	3.91e-34
2000	0.012	0.074	0.005	2.55e-34	2.97e-34
5000	0.012	0.155	0.005	2.55e-34	1.40e-33
9000	0.012	0.241	0.005	2.55e-34	3.25e-34

Supplementary Table 5: Average population frequency (f) of cluster (c), reference (r) and genomic (g; i.e. non-cluster and non-reference) insertions during a TE invasion. Only genomic insertions were simulated to have a negative impact on the fitness of the host ($x = 0.01$). Reference and cluster insertions were simulated neutral ($x = 0.0$). The significance of the allele frequency difference across 100 replicates was computed with a Wilcoxon rank sum tests. Note that cluster insertions consistently have a significantly higher frequency than reference insertions. Hence, cluster insertions are positively selected under this model. gen. generation

gen.	f_c	f_r	f_g	$p_{c\leftrightarrow r}$	$p_{c\leftrightarrow g}$
100	0.011	0.008	0.005	1.19e-19	2.56e-34
200	0.035	0.023	0.010	1.07e-20	2.55e-34
300	0.063	0.044	0.013	1.28e-13	2.55e-34
400	0.091	0.064	0.015	1.23e-12	2.63e-34
500	0.115	0.086	0.015	4.42e-08	2.56e-34
600	0.142	0.108	0.015	4.97e-07	5.74e-34
800	0.185	0.162	0.012	2.73e-03	2.42e-34
1000	0.229	0.194	0.004	9.76e-04	1.71e-35
1500	0.336	0.255	0.001	7.16e-05	3.05e-37
2000	0.424	0.312	0.001	2.95e-04	5.81e-38
5000	0.755	0.497	0.000	1.07e-03	2.63e-37
9000	0.910	0.557	0.000	4.88e-07	1.09e-37

References

Brian Charlesworth and Deborah Charlesworth. The population dynamics of transposable elements. *Genetical Research*, 42(01):1–27, 1983.
